# Supplementary material for: Pre-Operative Evaluation of DNA Methylation Profile in Oral Squamous Cell Carcinoma Can Predict Tumor Aggressive Potential
Source: Int J Mol Sci. 2020 Sep 14;21(18):6691. doi: 10.3390/ijms21186691 (PMC7555204; doi:10.3390/ijms21186691)
Supplement: Supplementary file 1 [file ijms-21-06691-s001.pdf]

Supplementary Materials

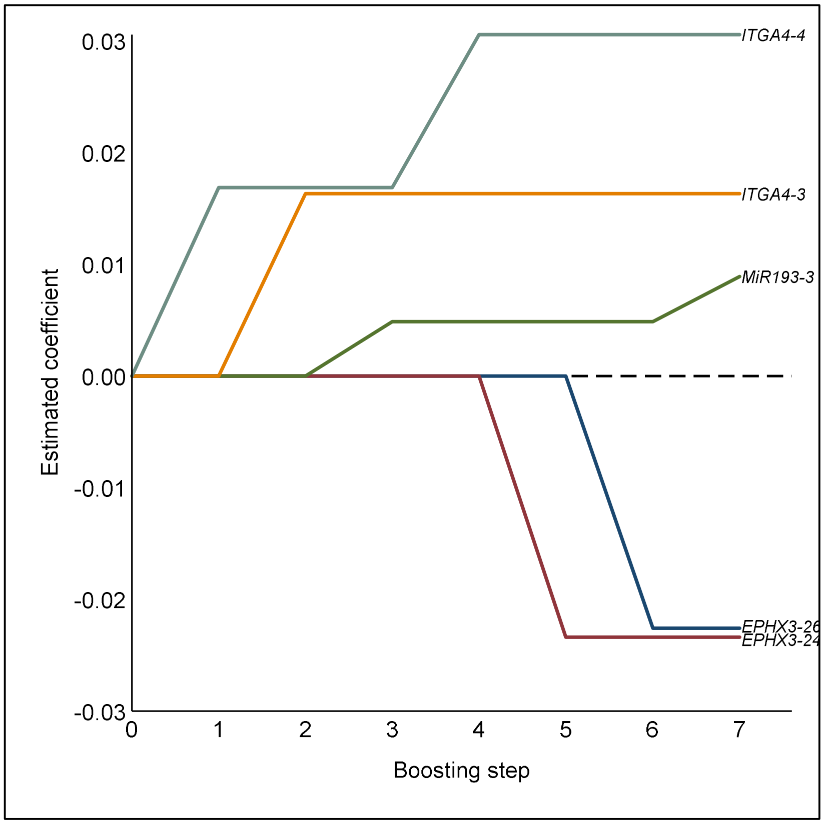

Figure 1. Coefficient paths of the five CpG sites included in the final Cox proportional hazards model. The coefficient estimates are plotted against the boosting steps as obtained from the likelihood-based component-wise algorithm. Coefficients are scaled to be at the level of the original methylation Beta-values.

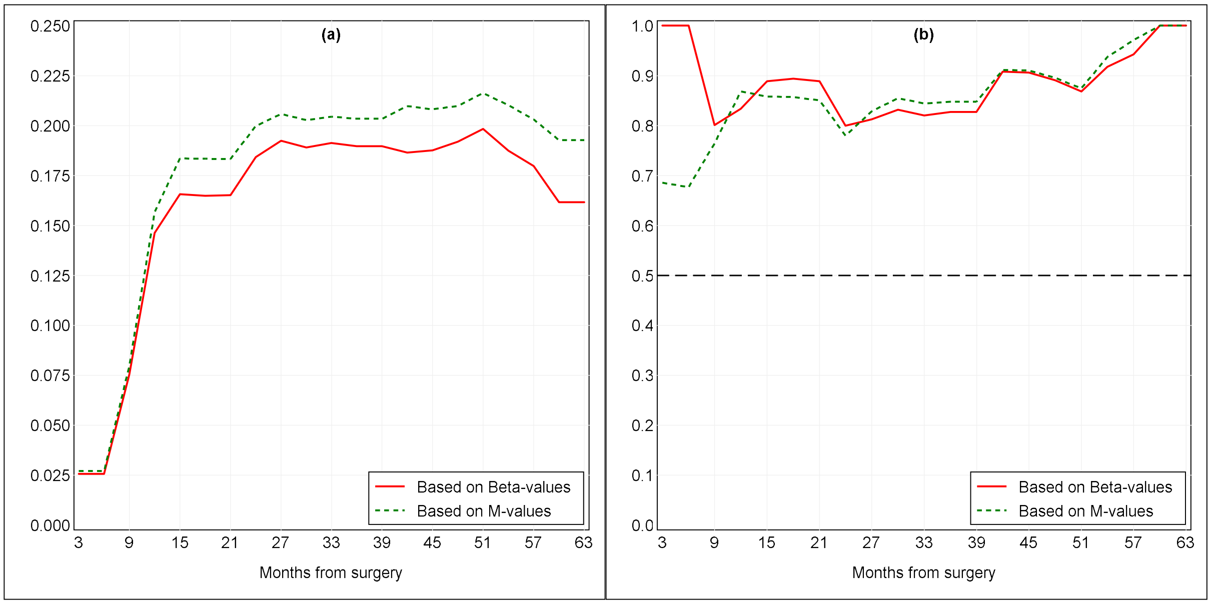

Figure S2. Time-dependent Brier score (a) and area under the receiver operator characteristic curve (AUC) (b) for the final Cox proportional hazards model fitted by component-wise likelihood-based boosting. The dashed line represents a non-discriminatory model (random guessing). M-values were computed as the binary logarithm of the intensities of methylated probes versus non-methylated probes. Performance and predictive accuracy of the model were estimated using the original training set of 36 patients.

Table S1. Permutation-based *P*-value estimations for all candidate methylation data (238 CpG sites). Covariates are ordered from smallest to largest *P*-value.

CpG site	*P*-value
ITGA4-4	0.0027
ITGA4-3	0.0078
MiR193-3	0.0099
EPHX3-24	0.0157
EPHX3-26	0.0172
EPHX3-23	0.0196
ITGA4-1	0.0234
EPHX3-18	0.0240
EPHX3-20	0.0268
MiR296-10	0.0286
TERT-2	0.0326
ITGA4-2	0.0350
ITGA4-5	0.0404
ITGA4-9	0.0411
EPHX3-21	0.0427
ITGA4-7	0.0451
EPHX3-12	0.0455
EPHX3-17	0.0476
EPHX3-16	0.0495
ITGA4-6	0.0511
TERT-1	0.0555
ITGA4-8	0.0578
EPHX3-25	0.0629
MiR296-14	0.0747
EPHX3-4	0.0766
ITGA4-12	0.0800
MiR296-9	0.0831
EPHX3-14	0.0878
ITGA4-11	0.0888
ITGA4-10	0.0921
EPHX3-22	0.0947
EPHX3-28	0.1005
EPHX3-2	0.1084
ITGA4-14	0.1151
EPHX3-5	0.1257
GP1BB-18	0.1270
EPHX3-10	0.1311
ITGA4-13	0.1439
EPHX3-15	0.1526
EPHX3-13	0.1570
FLI1-4	0.1646
EPHX3-8	0.1838
EPHX3-11	0.1859
EPHX3-3	0.1942
ZAP70-3	0.2019
MiR296-8	0.2043
FLI1-5	0.2044
ZAP70-1	0.2079
GP1BB-15	0.2102
EPHX3-6	0.2151
FLI1-8	0.2152
PARP15-1	0.4634
PARP15-15	0.4635
EPHX3-29	0.4638
MiR193-18	0.4720
LRRTM1-10	0.4733
MiR296-7	0.4804
MiR193-22	0.4821
PARP15-19	0.4854
ZAP70-9	0.4887
TERT-5	0.5093
EPHX3-7	0.5105
GP1BB-3	0.5157
NTM-4	0.5166
NTM-5	0.5187
ZAP70-17	0.5277
ZAP70-14	0.5418
GP1BB-17	0.5450
MiR296-15	0.5481
LRRTM1-3	0.5496
NTM-14	0.5572
ZAP70-15	0.5578
PARP15-9	0.5698
MiR193-25	0.5704
NTM-11	0.5734
NTM-3	0.5735
GP1BB-8	0.5813
TERT-3	0.5829
ZAP70-18	0.5844
ZAP70-8	0.5846
PARP15-5	0.5849
GP1BB-12	0.5884
GP1BB-11	0.5920
MiR296-6	0.5962
ZAP70-11	0.5963
LRRTM1-2	0.6014
ZAP70-12	0.6058
PARP15-13	0.6058
LRRTM1-11	0.6077
NTM-12	0.6098
NTM-10	0.6101
GP1BB-13	0.6184
NTM-1	0.6185
MiR296-1	0.6238
PARP15-6	0.6246
PARP15-10	0.6255
GP1BB-4	0.6269
MiR193-23	0.6295
LRRTM1-13	0.6313
NTM-9	0.6337
GP1BB-5	0.6341
MiR193-20	0.6383
GP1BB-7	0.7680
KIF1A-6	0.7686
KIF1A-20	0.7732
MiR296-3	0.7737
LINC0059-2	0.7751
LRRTM1-18	0.7780
KIF1A-3	0.7864
LINC0059-19	0.7889
MiR296-13	0.7965
NTM-15	0.8033
LRRTM1-16	0.8086
KIF1A-14	0.8093
LRRTM1-21	0.8108
KIF1A-26	0.8114
LINC0059-4	0.8136
LRRTM1-8	0.8177
PARP15-3	0.8233
LRRTM1-1	0.8245
PARP15-17	0.8251
KIF1A-22	0.8268
MiR296-12	0.8280
LRRTM1-22	0.8301
KIF1A-12	0.8306
MiR193-8	0.8331
LRRTM1-17	0.8351
LRRTM1-19	0.8377
KIF1A-8	0.8411
MiR193-4	0.8419
MiR193-24	0.8423
LINC0059-7	0.8429
MiR193-19	0.8432
MiR193-5	0.8452
LINC0059-15	0.8499
KIF1A-23	0.8525
TERT-4	0.8543
MiR193-11	0.8573
LINC0059-13	0.8635
KIF1A-25	0.8646
LRRTM1-4	0.8683
LINC0059-3	0.8739
KIF1A-27	0.8751
MiR193-14	0.8755
MiR193-17	0.8756
LINC0059-6	0.8822
KIF1A-1	0.8872
LINC0059-16	0.8888
MiR193-21	0.8938
LINC0059-14	0.8938
LRRTM1-20	0.8959
TERT-6	0.8997
PARP15-12	0.9038

EPHX3-1	0.2245
EPHX3-19	0.2249
MiR193-1	0.2291
MiR193-2	0.2408
ZAP70-4	0.2448
ZAP70-20	0.2449
GP1BB-1	0.2457
FLI1-2	0.2493
ZAP70-7	0.2696
ZAP70-5	0.2987
MiR296-5	0.3060
EPHX3-9	0.3064
PARP15-8	0.3078
FLI1-3	0.3314
ZAP70-2	0.3380
ZAP70-19	0.3542
MiR193-7	0.3586
FLI1-1	0.3613
EPHX3-27	0.3950
FLI1-7	0.4166
PARP15-2	0.4192
GP1BB-2	0.4205
ZAP70-16	0.4322
NTM-6	0.4449
ZAP70-6	0.4470
NTM-2	0.4508
GP1BB-14	0.4545
MiR296-11	0.4580
MiR193-10	0.4625
NTM-13	0.6433
MiR193-26	0.6471
GP1BB-9	0.6609
GP1BB-10	0.6669
MiR296-4	0.6688
NTM-7	0.6735
GP1BB-6	0.6745
LRRTM1-5	0.6773
MiR193-16	0.6790
LINC0059-5	0.6813
PARP15-18	0.6904
ZAP70-10	0.6918
LRRTM1-6	0.7063
LINC0059-18	0.7180
MiR193-9	0.7213
KIF1A-16	0.7251
KIF1A-5	0.7267
MiR193-15	0.7278
LRRTM1-12	0.7339
MiR193-13	0.7360
LINC0059-1	0.7434
KIF1A-2	0.7456
PARP15-16	0.7515
PARP15-14	0.7570
GP1BB-16	0.7573
KIF1A-9	0.7578
NTM-8	0.7583
PARP15-11	0.7603
LINC0059-20	0.7622
MiR193-12	0.9053
LRRTM1-9	0.9065
PARP15-4	0.9077
KIF1A-19	0.9104
KIF1A-11	0.9121
LINC0059-9	0.9164
KIF1A-18	0.9180
LRRTM1-15	0.9207
LINC0059-17	0.9239
LRRTM1-14	0.9253
LINC0059-8	0.9267
MiR296-2	0.9290
LINC0059-11	0.9304
ZAP70-13	0.9321
LINC0059-12	0.9366
MiR193-6	0.9426
LINC0059-10	0.9448
KIF1A-15	0.9475
PARP15-7	0.9476
KIF1A-17	0.9559
LRRTM1-7	0.9565
KIF1A-4	0.9586
KIF1A-10	0.9608
KIF1A-24	0.9674
KIF1A-21	0.9674
KIF1A-13	0.9689
KIF1A-7	0.9760

Table S2. Cox proportional hazards model resulting from likelihood-based component-wise boosting with no information on connection among same-gene CpG sites (optimal number of steps = 10), and after conversion of Beta-values into M-values (optimal number of steps = 4). Non-zero regression coefficients (ln HR) are presented along with hazard ratios (HRs) and permutation-based *P*-values. Coefficients are scaled to be at the level of the original methylation data.

CpG site	Beta-values, no pathway connection information		M-values*	
	Ln HR	HR	Ln HR	HR
EPHX3-18	·	·	–0.3940	0.6744
EPHX3-23	–0.0195	0.9807	·	·
EPHX3-24	–0.0235	0.9768	·	·
EPHX3-25	·	·	–0.4256	0.6534
ITGA4-4	0.0577	1.0594	0.1884	1.2073
MiR193-3	0.0176	1.0178	0.2192	1.2451
Integrated Brier score	*0.089*		*0.070*	
C-index	*0.805*		*0.806*	
Integrated AUC	*0.847*		*0.834*	

* Pathway connection information had no impact on variable selection.
